# Nationwide cross-sectional study results on long-term care and SARS-CoV-2 infection among older adults in Germany during the COVID-19 pandemic

**DOI:** 10.1038/s41598-026-37108-7

**Published:** 2026-02-02

**Authors:** Ana Magdalena Ordonez-Cruickshank, Hannelore Neuhauser, Arina Zanuzdana, Christina Poethko-Müller, Beate Gaertner, Judith Fuchs

**Affiliations:** 1 Department of Epidemiology and Health Monitoring, Robert Koch Institute, Nordufer 20, 13353 Berlin, Germany; 2 Charité – Universitätsmedizin Berlin, corporate member of Freie Universität Berlin and Humboldt-Universität zu Berlin, Berlin, Germany; 3 Department of Infectious Disease Epidemiology, Robert Koch Institute, Berlin, Germany

**Keywords:** Aged, COVID-19, Long-term care, Home care, Risk factors, Diseases, Health care, Medical research, Risk factors

## Abstract

This study investigates determinants of self-reported SARS-CoV-2 infection, addressing a gap in understanding how social and care-related factors are associated with infection risk among older people. We analyzed the baseline wave of “Gesundheit 65+”, a German nationwide population-registry survey. This wave was performed between June 2021 and April 2022, with a sample of 3450 participants aged 65 to 100 living in private households. SARS-CoV-2 infection prevalence was 3.5% (95% confidence interval, CI 2.6–4.5), slightly higher among those aged 85+, and higher among those receiving home care, and not having double vaccination. We identified with multivariable logistic regression that not having double vaccination (odds ratio, OR = 9.72; CI 4.81–19.61), receiving in-person visits (OR = 2.96; CI 1.12–7.80), and not living alone (OR = 1.96; CI 1.02–3.76) were independently associated with SARS-CoV-2 infection, but receiving formal or informal care at home, and participating in work and social activities were not. Our results emphasize the importance of vaccination and caution during in-person interactions. Despite receiving formal or informal care at home implying close personal contact, we did not detect increased infection risk in those receiving care at home.

**Supplementary Information:**

The online version contains supplementary material available at 10.1038/s41598-026-37108-7.

## Introduction

At the beginning of the COVID-19 pandemic in Europe, with no vaccines available, older people were especially vulnerable to infection and experienced the worst outcomes^1^. In Germany, people 60 years and older had the highest rates of hospitalization and mortality during the first COVID-19 wave in 2020^2,3^. Throughout the pandemic, various risk factors for SARS-CoV-2 infection have been identified in both younger and older adults. A systematic literature review conducted in 2022 pinpointed several factors contributing to infection risk, including for example older age, and lower levels of education^4^. People in urban settings and larger households faced increased risk^4,5^ and in-person visits also elevated the likelihood of infection^6^. Health-related factors such as multimorbidity and obesity further increased these risks, raising the chance of contracting the virus and leading to more severe outcomes once infected^4,6,7^.

Moreover, the pandemic significantly affected residents of long-term care facilities (LTCF), who were particularly susceptible^8,9^. However, the provision of long-term care is not exclusively limited to LTCFs. In 2021, 4.96 million people in Germany received long-term care due to limitations in their basic activities of daily living (ADL)^10^; more than 79% were over the age of 65, and 84% were living in the community^10^. Among those receiving care at home, 51% received informal care only - defined as care provided by family, close relatives, friends, or neighbors - while 21% received home care - defined as receiving support at home from an outpatient nursing service^10^. However, there is a lack of studies focusing on older community-dwelling individuals during the pandemic reporting data on SARS-CoV-2 infection, vaccination rates and associated factors^11^, especially for those needing long-term care due to morbidity or frailty.

By June 2021, when the study commenced, the vaccination campaign had been running for six months; 50.1% of the population in Germany had received at least one of the two recommended doses of a COVID-19 vaccine^12^. The Standing Committee on Vaccination (STIKO) in Germany recommended having basic immunization through vaccination^13^. Basis immunity was defined as at least two SARS-CoV-2 antigen contacts, achieved through exposure to antigens via infection or vaccination. According to official data, at the turn of 2021/2022, approximately 70% of adults older than 60 had received basic immunization^14,15^. Vaccination campaigns prioritized older people, especially those living in LTCFs^16,17^. From March 2021, testing centers were available, offering people the opportunity to receive one free rapid antigen test per week. If the rapid antigen test was positive, it had to be confirmed by a polymerase chain reaction (PCR) test^18^. Rigorous testing schemes were applied to people living, visiting or working in nursing homes^19,20^. This resulted in infections being detected more frequently among nursing home residents than among older people living in the community.

When the study started, the third wave of the pandemic was ending, and during the summer of 2021 there was a lower incidence of SARS-CoV-2 infections^14^. The predominant variant of coronavirus was the Delta variant^21^. Summer 2021 was followed by a surge of new infections during the fourth wave of the pandemic in Germany, from November 2021 to the end of April 2022, due to the emergence of the Omicron variant of SARS-CoV-2^21^. Nevertheless, lockdowns and other restrictive measures were gradually lifted thanks to rapid testing and vaccination campaigns, allowing for more in-person interaction.

The Robert Koch Institute (RKI), Germany’s national institute for public health, has conducted the population-representative Study on Health of Older People in Germany, “Gesundheit 65+”, to assess the health of older and very old people during the pandemic in Germany^22^. In this analysis of Gesundheit 65+, we aim to describe the prevalence of self-reported SARS-CoV-2 infection in the older population in Germany living in private households and identify the most vulnerable groups to SARS-CoV-2 infection.

## Results

### Sample description

A total of 3694 people aged 65 to 100 participated in the survey, from whom 3450 living in private households were included in the analysis. The weighted average age was 75.4 years (standard deviation = 7.4 years); 49.7% of the participants were 65 to 74, and 12.4% were 85 or older. Females comprised 55.3% of the sample. Table 1 shows the characteristics of the participants. Most participants had a low level of education (54.2%) and lived in small towns (34.6%). The majority of participants reported multimorbidity (71.1%), had received in-person visits (89.6%), did not live alone (68.8%), and participated in work and/or social activities (60.1%). Participants less often reported risk factors such as receiving informal support at home (25.6%), smoking (10.1%), not having double vaccination (7.9%), or receiving long-term home care (5.4%).

**Table 1:** Characteristics of the total population of the study Gesundheit 65+ living in private households by infection status, weighted column % (95% CI), unweighted n.

	Total			Positive test for SARS-CoV-2 infection		No SARS-CoV-2 infection
	*n*	% (95% CI)	*n*	% (95% CI)	*n*	% (95% CI)
**Total**	3450	100	114	3.5 (2.6–4.5)	3336	96.5 (95.5–97.4)
**Sociodemographic risk factors**						
Age group in years						
65–74	1095	49.7 (47.4–52.0)	38	50.1 (37.6–62.7)	1057	49.7 (47.5–52.0)
75–84	1680	37.9 (35.6–40.2)	50	34.2 (23.5–46.7)	1630	38.0 (35.7–40.4)
85+	675	12.4 (11.1–13.8)	26	15.7 (8.3–27.5)	649	12.3 (11.1–13.6)
Sex						
Female	1630	55.3 (54.0–56.5)	58	56.7 (45.5–67.3)	1572	55.2 (53.8–56.6)
Level of education						
Low	1654	54.2 (50.9–57.5)	58	51.3 (37.6–64.8)	1596	54.3 (51.0–57.6)
Middle	931	31.6 (29.0–34.4)	27	32.2 (20.7–46.3)	904	31.6 (29.0–34.3)
High	819	14.2 (12.6–15.9)	28	16.5 (9.1–28.1)	791	14.1 (12.5–15.9)
Municipality size						
Rural	482	16.2 (10.5–24.3)	11	11.4 (5.0–23.9.0.9)	471	16.4 (10.6–24.5)
Small town	1212	34.6 (26.4–43.7)	41	39.4 (25.0–56.0)	1171	34.4 (26.3–43.5)
Medium town	892	24.0 (17.3–32.4)	23	19.2 (9.1–35.9)	869	24.2 (17.4–32.6)
City	864	25.2 (18.1–34.0)	39	30.0 (17.0–47.2.0.2)	825	25.0 (17.9–33.8)
**Health related risk factors**						
Multimorbidity	2470	71.1 (68.7–73.4)	92	78.5 (64.1–88.2)	2378	70.9 (68.5–73.1)
Smoking	255	10.1 (8.6–11.9)	6	3.8 (1.2–11.2)	249	10.3 (8.8–12.2)
**Personal contact related risk factors**						
Type of support						
No support	2085	69.0 (66.5–71.5)	59	63.9 (49.9–75.8)	2026	69.2 (66.6–71.7)
Informal support	942	25.6 (23.4–28.0)	37	27.8 (17.7–40.7)	905	25.5 (23.2–28.0)
Home care	229	5.4 (4.5–6.4)	10	8.3 (2.9–21.4)	219	5.2 (4.4–6.3)
Not living alone	2247	68.8 (66.4–71.2)	75	76.9 (65.4–85.5)	2172	68.6 (66.1–70.9)
Participated in work/social activities	1878	60.1 (57.3–62.8)	57	56.1 (43.2–68.3)	1821	60.2 (57.5–62.8)
Received in-person visits	3027	89.6 (88.1–91.0)	101	93.3 (85.6–97.0)	2926	89.5 (87.9–90.9)
**Immunization**						
Not having double vaccination	212	7.9 (6.3–9.9)	29	33.1 (20.6–48.5)	183	7.0 (5.5–8.9)

Notes: Observations with missing information on education 46; smoking 49; type of support 194; living alone 87; participated in work/social activities 62; received in-person visits 63; vaccination 69; 95% CI = 95% confidence interval.

### Self-reported SARS-CoV-2 infection

A total of 114 people reported ever having a positive SARS-CoV-2 test, corresponding to 3.5% of the participants living in private households. The participants differed depending on their infection status. More people aged 85+ reported having a positive test. People reporting a positive test had more often a higher level of education, lived in cities, had higher rates of multimorbidity, reported lower prevalence of smoking, received more often support at home, and a higher proportion of them reported living alone and received in-person visits compared to the group without an infection (Table 1). However, due to the large 95% confidence intervals in the group with positive test for infection, most confidence intervals overlap between the two groups. The only significant difference (seen by the non-overlapping confidence intervals (Table 1) and from bivariate logistic regression (Table 2)) found between the two groups was the lack of double vaccination, with a third (33.1%) of the participants with an infection reporting not having received double vaccination, compared to 7.0% of those without infection.

**Table 2:** Logistic regression analyses of factors associated with self-reported SARS-CoV-2 infections at the time of the baseline wave of the Gesundheit 65+ study among participants living in private households.

	Bivariate analysis		Multivariable analysis (*n* = 3061)	
	OR (95% CI)	*p*-value*	OR (95% CI)	*p*-value*
**Sociodemographic risk factors**				
Age group in years (ref. 65–74)				
75–84	0.89 (0.51–1.55)	0.686	1.08 (0.59–1.98)	0.810
85+	1.27 (0.60–2.68)	0.537	1.13 (0.53–2.42)	0.750
Sex (ref. male)				
Female	1.06 (0.66–1.71)	0.799	1.07 (0.64–1.80)	0.798
Level of education (ref. high)				
Low	0.80 (0.38–1.69)	0.563	0.78 (0.35–1.75)	0.547
Middle	0.87 (0.40–1.88)	0.716	0.88 (0.38–2.00)	0.750
Municipality size (ref. rural)				
Small town	1.65 (0.74–3.67)	0.216	1.99 (0.80–4.95)	0.139
Medium town	1.14 (0.44–2.96)	0.784	1.17 (0.43–3.20)	0.761
City	1.73 (0.74–4.01)	0.202	2.32 (0.89–6.02)	0.083
**Health related risk factors**				
Multimorbidity (ref. no multimorbidity)	1.50 (0.75–3.02)	0.253	1.54 (0.74–3.22)	0.250
Smoking (ref. not smoking)	0.34 (0.11–1.12)	0.076	0.35 (0.10–1.22)	0.098
**Personal contact related risk factors**				
Type of support (ref. no support)				
Informal support	1.18 (0.63–2.20)	0.603	1.13 (0.56–2.25)	0.733
Home care	1.72 (0.55–5.35)	0.347	1.58 (0.58–4.29)	0.365
Not living alone (ref. living alone)	1.53 (0.87–2.68)	0.139	1.96 (1.02–3.76)	0.042
Participated in work/social activities (ref. no work/social activities)	0.85 (0.51–1.40)	0.509	0.86 (0.51–1.44)	0.559
Received in-person visits (ref. no in-person visits)	1.62 (0.70–3.78)	0.261	2.96 (1.12–7.80)	0.028
**Immunization**				
Not having double vaccination (ref. double vaccination)	6.56 (3.41–12.62)	< 0.001	9.72 (4.81–19.61)	< 0.001

Notes: *Wald test; OR = odds ratio; 95% CI = 95% confidence interval; ref. = reference group.

**Fig. 1:**
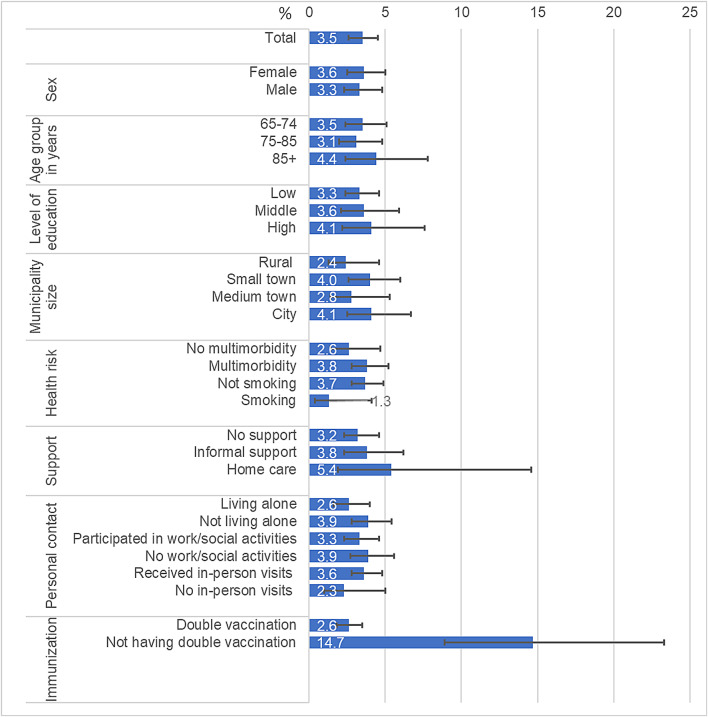
Prevalence of past self-reported SARS-CoV-2 infections at the baseline wave of the Gesundheit 65+ study among participants living in private households.

The prevalence of SARS-CoV-2 infection by risk factors is shown in Fig. 1. The highest prevalence was seen for those without double vaccination (14.7%), those reporting home care (5.4%), and those in the oldest age group (4.4%). The lowest rates were reported for those smoking (1.3%), those not receiving in-person visits (2.3%), and living in rural areas (2.4%).

### Association of SARS-CoV-2 infection with sociodemographic, health, personal contact, and COVID-19 pandemic risk factors

In the multivariable logistic regression model, we included all risk factors as test for multicollinearity showed low correlations between variables (Supplementary table S1). Not living alone, receiving in-person visits and not having double vaccination were independently associated with SARS-CoV-2 infection. After adjusting for other variables in the model, the highest odds for infection were found amongst participants without double vaccination (odds ratio, OR = 9.72; 95% confidence interval, CI 4.81–19.61), followed by participants receiving in-person visits (2.96; 1.12–7.80), and not living alone (1.96; 1.02–3.76).

We additionally characterized infected participants by type of support (Table 3). Because there was only a low number of infected participants, we collapsed those who received support at home (i.e. informal support or home care) into one group for this analysis. In a descriptive analysis, infected participants differed depending on the type of support received. Infected participants living at home without support were more often aged 65–74 years, tended to be less often women, tended to have higher levels of education, and more of them participated in work and social activities compared to participants living at home and receiving support. Furthermore, individuals receiving support at home reported less often not having double vaccination than infected participants who did not receive support. Approximately the same proportion of participants in both groups reported not living alone and receiving in-person visits.

**Table 3:** Characteristics of the infected participants at the baseline wave of the Gesundheit 65 + study among participants living in private households by type of support - weighted column % (95% CI).

	No support	Support at home (= informal support or home care)
	*n* = 59	*n* = 47
**Sociodemographic risk factors**		
Age group in years		
65–74	64.6 (48.3–78.1)	24.5 (11.2–45.7)
75–84	30.3 (17.7–46.7)	44.9 (25.7–65.7)
85+	5.2 (1.8–14.0)	30.5 (14.0–54.3)
Female sex	43.8 (29.0–59.7)	77.5 (63.1–87.4)
Level of education		
Low	39.7 (24.4–57.3)	68.1 (42.6–86.0)
Middle	36.5 (21.5–54.6)	26.6 (10.3–53.3)
High	23.8 (12.1–41.4)	5.3 (1.7–15.6)
**Personal contact related risk factors**		
Not living alone	78.2 (62.6–88.5)	75.7 (57.9–87.6)
Participated in work/social activities	71.4 (55.4–83.4)	28.2 (14.6–47.4)
Received in-person visits	95.1 (85.8–98.4)	93.1 (81.3–97.7)
**Immunization**		
Not having double vaccination	38.4 (21.8–58.2)	26.7 (10.8–52.3)

Notes: 95% CI = 95% confidence interval; unweighted n; missing information on support for infected participants *n* = 8.

## Discussion

In this nationwide population-based study among people between 65 and 100 years of age conducted between June 2021 and April 2022, 3.5% of the participants reported a previous infection of SARS-CoV-2. By that time, prevalence of infection was highest among those reporting not having double vaccination and receiving home care. In contrast, those not receiving in-person visits reported the second lowest SARS-CoV-2 prevalence rates (only after smokers). These results were also confirmed by multivariable analyses adjusted for various risk factors.

The prevalence of SARS-CoV-2 infection and double vaccination in our study (3.5% and 92.1%, respectively) were similar to the prevalence of infections reported in other national studies and by the RKI. The RKI reported a prevalence of SARS-CoV-2 infection of 3.4% in June 2021 in people aged 60 years and older^16^, and the prevalence of self-reported infection found in the second Corona Monitoring Nationwide (RKI-SOEP-2) Study was 4.0%^14^. The RKI-SOEP-2 study was completed at the end of 2021 and beginning of 2022, approximately in the same period as the baseline wave of Gesundheit 65+. The prevalence of double vaccination status in our study (92.1%) was similar to the prevalence reported by the German Ministry of Health for people aged 60 years and older in 2023 (91.1%)^23^ but lower than the prevalence of vaccination in RKI-SOEP-2 for people aged 65 years and older (95.9%)^14^.

Our findings revealed that not having double vaccination against COVID-19 increased the odds of having a SARS-CoV-2 infection by more than nine times compared with those who had received double vaccination, contributing further to the evidence of vaccine effectiveness in older people^24^. However, at the time of the study it was recommended to wait six months after a confirmed SARS-CoV-2 infection before receiving the corresponding vaccine dose^25^. As we did not have information on the date of infection and vaccination, it is possible that some participants actually had a previous infection and were recommended not to receive the vaccine, thus appearing to not have double vaccination. Therefore, this analysis has possibly overestimated the risk of not having double vaccination. Consequently, we performed a sensitivity analysis stratifying by date of participation in which immunization remained a significant factor that increases the odds for infection between June to December 2021 and January to April 2022 (Supplementary table S2). We also performed a second sensitivity analysis with a different definition for immunization. In this analysis, not having any dose of the vaccine was still significantly associated with infection and increased the odds of infection from three to over nineteen times (Supplementary table S3).

Identifying risk factors for infection in the older population is crucial for developing an effective pandemic preparedness strategy. This is the first nationwide study in Germany on risk factors for SARS-CoV-2 infection specifically targeting community-dwelling older adults. It focuses on the associations between infection and social risk factors like personal contact. Our research, which investigates infections for the first two and a half years of the COVID-19 pandemic in Germany among individuals aged 65 years and older, enhances our understanding of how health status, types of support in activities of daily living, and social engagement may influence the risk of infection in older adults. We paid particular attention to the type of support these individuals receive, as this aspect has often been overlooked in previous research^26,27^.

An important strength of our study was our ability to describe the German older population according to support groups. The group receiving home care had the highest prevalence of SARS-CoV-2 infection compared with those living at home without support or receiving informal care. This is similar to what was found in a Swedish study including 3,410,241 participants, where those who received home care and residential care had the highest odds of contracting a SARS-CoV-2 infection compared with those who did not require care^28^. However, the study only described formal care and independent living; people receiving informal care were not differentiated. The higher infection prevalence in the group that received some kind of support at home has been explained by the fact that older adults in private households needing home care may have more difficulties accessing vaccines, and effectively following hygiene measures than independent older adults, probably because of mobility and cognitive problems^26,29,30^. Our results indicate that the group receiving support had slightly better vaccine coverage than the group of independent people, but these results were not statistically significant. In our multivariable model, receiving either home care or informal care was not significantly associated with increased odds of SARS-CoV-2 infection.

It is important to note that, at that point in the pandemic in Germany, when vaccines were already available, we found no evidence that work or other social activities posed a risk of infection in older people, at least not with the moderate statistical power of our study. An exception was in-person visits that increased the odds of infection compared to no visits, only during the early study period (Supplementary table S2). From June to December 2021, being 85+, having higher education and in-person visits were associated with higher odds of infection. During the second period of the study (January to April 2022), these factors were no longer associated with infection. Increasing overall vaccination coverage and awareness of the general population on a “cocoon strategy” for vulnerable older people - meaning the vaccination of people in close contact with this vulnerable group - as well as lockdown measures, may have contributed to this favorable result. In this regard, a Swedish register-based study showed that limiting in-person visits from persons outside the household and limiting social activities in people older than 70 was a protective factor for SARS-CoV-2 infection^31^. Additionally, living with other people in the same household increased the odds of infection compared with living alone. Cohabitation has previously been described as a risk factor for infection. In the context of people in need of home care or informal care at home, it is essential to develop further evidence on household transmission of the virus^32^. However, our calculated 95% confidence intervals for the estimated OR of receiving in-person visits and of not living alone were relatively wide. This indicates that, although the effect estimates suggest a relationship between in-person visits, cohabitation and infection (OR around 2), the confidence intervals are wide due to the small sample size and the fact that the associations changed in the course of the study period. While our analyses focus on risk of infection, living alone in a pandemic has relevant other aspects to consider such as social isolation and loneliness that lead to a higher risk of depression, anxiety, increased sedentary behavior and even cognitive decline^33–36^. Furthermore, a study using data from the Survey of Health, Ageing, and Retirement in Europe (SHARE) found that a higher frequency of face-to-face contact with adult children had a protective effect for SARS-CoV-2 infection in mothers and was not a risk factor for fathers, showing a protective effect of intergenerational social support during the second wave of the pandemic^37^.

Despite previous evidence showing that people living in bigger cities have a higher risk of infection due to socioeconomic factors and a higher population density, the municipality size was not significant in our analyses^38^. However, OR for people living in non-rural areas tended to be higher compared to people living in a rural area. Age or multimorbidity were not significantly associated with infection in our sample of people aged 65+. These results are similar to results from a study using South Korean National Health Insurance data, where older age and male sex were not found to be risk factors for SARS-CoV-2 infection but were rather risk factors for severe outcomes^39,40^. However, the same authors’ findings suggest that multiple advanced comorbidities are a risk factor for infection. In our study, we did not use a prognostic tool such as the Charlson Comorbidity Index, but we examined the coexistence of two or more chronic diseases without considering the stage of the diseases at the time of the study, which may explain the difference in our results. Also, the number of chronic conditions considered in the questionnaire is limited and perhaps including more or different chronic conditions (i.e. neurodegenerative diseases, chronic kidney disease, liver disease, asthma, or autoimmune diseases like rheumatoid arthritis) would influence the association between multimorbidity and infection. In addition, we may have underestimated the effect of multimorbidity or other risk factors due to survivorship bias, as the study was conducted in the second year of the pandemic, when older people with more advanced comorbidities may have succumbed to the virus.

Paradoxically, smoking participants had a lower prevalence of SARS-CoV-2 infection. Smoking also tended to have decreased odds for infection in the bivariate and multivariable analysis, despite the results not being statistically significant, and additional analyses stratified by study period showed that a decreased odd for infection was only present in the earlier study phase (Supplementary table S2). This phenomenon has been observed in other studies^41,42^. A social desirability bias could explain this finding, as we are working with self-reported data, which tend to underestimate smoking status^43^. In addition, there is evidence to suggest that the increased likelihood of contracting COVID-19 or having more severe outcomes due to smoking, led to more individuals quitting smoking or smokers adhering more to preventive hygiene measures^44^. Furthermore, due to increased severity of infection and higher mortality due to COVID-19, smokers with SARS-CoV-2 infection may have been missed or underrepresented in the study due to survivorship bias^45^. Still, there is evidence that suggests there are multiple mechanisms through which smoking reduces the risk of SARS-CoV-2 infection. For example the anti-inflammatory properties mediated by nicotinic acetylcholine receptors^46^, or the reduction of the angiotensin-converting enzyme 2 expression in bronchial cells^47^, which is essential for coronavirus infectivity. Nevertheless, these mechanisms are not fully understood. Tobacco is still an important cause of morbidity and mortality worldwide and its use should not be encouraged for the prevention of SARS-CoV-2 infection.

Some of the current study’s limitations are due to the nature of our cross-sectional analysis of the data, the causality between various factors and SARS-CoV-2 infection among older adults living in Germany cannot be ascertained, especially since we do not have information on the date of infection or vaccination. Also, there is no clear temporal association between infection and support needs, meaning long-term care needs may have arisen after a SARS-CoV-2 infection. The study excluded people with insufficient German language knowledge; therefore, the generalizability of our study might be limited regarding people with a migration background. Nevertheless, according to the German Federal Statistical Office, 77% of people living in Germany speak only German in the household, only 6% of the population living in Germany do not speak German in the household, and 76% of the people with migration background reckon their German knowledge as good or very good, which would allow these people to answer the Gesundheit 65+ study questionnaire^48^. This is in line with observation in our study, among the 12,248 people invited to participate in Gesundheit 65+, only 307 people had to be excluded from study participation^49^. Of those, 107 people were excluded due to insufficient German language proficiency, representing less than 1% of the unadjusted gross sample. Recall bias is always possible in studies with self-report, notably because of the prevalence of cognitive problems in this population. However, the possibility of having a proxy answering the questionnaire could diminish this bias. A major strength of the study Gesundheit 65+ is that it aimed to include participants with and without severe functional limitations living in the community, regardless of their living conditions, health or cognitive status. The study managed to include such hard-to-reach individuals through proxy participation and consent by legal representatives, as well as the mixed-mode survey design, which allowed participation via a paper-based, web-based, telephone, or face-to-face questionnaire/interview. Additionally, people aged 80 years and over, who are usually underrepresented in other population-based surveys were oversampled in this study. As a result, people who would normally be excluded from other studies were able to participate in Gesundheit 65+, adding to the representativeness of the study^49–51^.

In conclusion, our results reaffirm the effectiveness of vaccines in protecting older adults against SARS-CoV-2 infection. In-person social interaction with people older than 65 should be exercised with caution. Further research is needed to make in-person interactions safer for older people while not recurring to social isolation. Although non-care related in-person contact is a risk factor for infection, there was no indication that delivering formal or informal care to older adults in a private home setting was significantly associated with infection in older adults. However, it is necessary to perform further analyses to assess the causality between infection and the need of long-term care.

## Methods

### Study design and study participants

Gesundheit 65+ is a population-based longitudinal study performed from June 2021 to April 2023 to provide information on the health and well-being of the population aged 65 years and older in Germany during the COVID-19 pandemic. This analysis only considers the baseline wave, that was conducted from June 2021 to April 2022. Sample selection of Gesundheit 65+ followed a two-stage stratified cluster sampling procedure whereby 128 randomly selected primary sampling units (PSUs) were drawn from all municipalities in Germany and then stratified based on region and the BIK-10 classification^52^, a regional classification system for Germany. Random samples were drawn from the respective population registries in Germany, stratified by sex and two age groups (65–79 years and 80 years and older)^22^. Individuals 65 years or older who were permanently residing in Germany were the target population of our study. Participants could complete the survey in written form via paper or an online questionnaire, or via a telephone or face-to-face interview. Proxy information, e.g. via family members, was allowed. Invitees were only excluded if they had insufficient German language proficiency, had died or moved away before the fieldwork period started, or could not be located.

A total of 3694 individuals participated in the baseline survey, corresponding to a response rate of 30.9%^49^. To evaluate risk factors for SARS-CoV-2 infection, our analysis was restricted to participants with complete information on infection status assessed by the question, “Have you ever been infected by the Coronavirus (SARS-CoV-2)?” The possible answers were “Yes, confirmed by a test”, “Yes, probably, but not confirmed by a test,” or “No”. Until January 2022, all positive rapid antigen tests for SARS-CoV-2 infection had to be confirmed by a PCR test in Germany. Only afterwards it was recommended to rely on antigen tests alone^53^. Therefore, infection in our sample corresponds to either a positive PCR or a rapid antigen-test at any time prior to the study participation, including times before vaccines were available. In total 93 participants had to be excluded from our analysis because their infection was not confirmed by a test or because information on this question was missing. Since there were different vaccination and testing measures for people living in LTCF^16^, we further restricted our analysis to people living in private households. Therefore, further 151 participants who reported living in “special housing for older people”, or “nursing home or assisted living facility” were excluded. This left a total of 3450 participants for our analysis.

### Ethical considerations

The Federal Ministry of Health funded the study (grant no: ZMVI1-2518FSB410), and its implementation was approved by the Institutional Data Protection Officer and by the Ethics Committee of the Berlin Medical Association (Eth- 50/19). The study protocol describes the study design in detail^22^. The study participants or their legal representatives signed a written informed consent during the baseline survey. Study participants in the online survey gave their consent online. The study was conducted in accordance with the Declaration of Helsinki. All methods were carried out in accordance with relevant guidelines and regulations.

## Measurements

### Immunization

Vaccination against COVID-19 was operationalized using the question “Have you already been vaccinated against the coronavirus disease (COVID-19)?” with the possible answers “Yes, once”, “Yes, twice” or “No.” This variable was dichotomized into being vaccinated twice (yes/no)^16^.

### Health-related risk factors

To study multimorbidity, we used the 12-month self-reported prevalence of the following chronic diseases based on a list according to the European Health Interview Survey (EHIS)^54^: hypertension (high blood pressure), coronary heart disease (incl. myocardial infarction or chronic symptoms secondary to myocardial infarction, angina pectoris), stroke (incl. chronic symptoms secondary to a stroke), hypercholesterolemia (high blood lipids), diabetes, chronic bronchitis (incl. chronic obstructive pulmonary disease, emphysema), arthrosis, osteoporosis, lower back disorder or other chronic back defect, and depression. Additionally, cancer and obesity were included. Cancer was recorded by asking “Has a doctor ever diagnosed you with cancer?” Self-reported information on weight and height was used to calculate the body mass index (BMI). Obesity was defined as a BMI > = 30 kg/m^2^ (yes/no)^55^. The total sum of the prevalent diseases and health problems was calculated from the responses (range: 0 to 12). Multimorbidity was defined as the co-occurrence of two or more of the twelve abovementioned conditions (yes/no)^56^, allowing for up to 11 missing values. We assumed that in the chosen list format, diseases that the participants were not suffering from or were unaware of often went unanswered.

Smoking was defined as current daily or occasional smoking and not smoking anymore or never having smoked was considered not smoking.

### Personal contact-related risk factors

As face-to-face contacts that could have increased the risk of a SARS-CoV-2 infection, we considered contacts due to (a) receiving care or support related to limitations to perform basic or instrumental ADL, (b) living situation, and (c) work and social activities.

Three mutually exclusive types of support (home care, informal care at home, and no support) were defined. When participants reported difficulties performing any of twelve basic or instrumental ADL^57,58^, they were asked if they usually had help with any of these activities; if yes, information on the care giving persons was collected. If participants received support from an outpatient nursing service, they were classified as receiving home care. If participants received support only from a person within the same household or another person, they were classified as receiving informal support at home. All other participants were classified as receiving no support.

Participants reported the number of people living in the same household. This variable was dichotomized into “living alone” (yes/no).

Social and work activities were examined by asking the participants about their in-person participation in the last month according to an adapted list of activities^59^ including: religious events, cultural or educational activities, paid work, voluntary work, or childcare (each: yes/no). If a positive response was received for at least one of the activities, the respondent was considered to have participated in a work or social activity (yes/no), allowing for up to four missing values. Family or friend visits in the last month were studied as a separate variable as in-person visits (yes/no).

### Sociodemographic risk factors

The demographic variables comprised sex and age in years at the time of the baseline survey, described in three age groups (65–74, 75–84, and 85 years or older) reflecting the different stages of aging aligning with many public health policies^60^. In addition, level of education was measured using the Comparative Analysis of Social Mobility in Industrial Nations (CASMIN) scale which categorizes participants into one of three groups: (1) low education (i.e. primary and low secondary education), (2) medium education (i.e. intermediate/high secondary education), and (3) high education (i.e. tertiary education) based on the highest level of education obtained^61^. Municipality size was categorized as rural (population < 5,000), small town (population 5,000 to < 20,000), medium town (population 20,000 to < 100,000), and city (population 100,000 and more).

### Statistical analyses

A weighting factor was calculated to adjust the prevalence for deviations of the sample from the target population of people aged 65 and over in Germany as of December 31, 2020, in terms of sex, age, region and municipality size according to the BIK-10 classification^52,62^. Deviations in the level of education compared to the resident population of Germany based on the 2018 micro-census according to the International Standard Classification for Education (ISCED classification) were also considered to calculate the weighting factor^62,63^. In order to adequately take into account the grouping by PSUs and the weighting in the calculation, all analyses were carried out using survey procedures. Descriptive statistics were used to summarize the participant characteristics as weighted percentages (%) and 95% confidence interval by infection status 95%. We also described the prevalence of SARS-CoV-2 infection stratified by the variables of interest. We conducted bivariate logistic regressions using Wald tests to explore the crude associations between SARS-CoV-2 infection and variables of interest. All studied variables were used in a multivariable logistic regression model to evaluate the associations between different risk factors and infection. All analyses were performed using Stata/SE 17.0 (Stata Corp., College Station, TX, USA, 2017) with a significance level *p* < 0.05.

### Sensitivity analyses

To assess the consistency of our results throughout the baseline wave (June 2021 to April 2022), we considered December 2021 as a turning point of the pandemic since the Omicron variant became more prevalent by the end of this month^64^. People reporting infection before January 2022 were probably infected by the Alpha or Delta variant. In contrast, people who answered the questionnaire after December 2021 and reported an infection could be affected by the Alpha, Delta, or Omicron variants. Therefore, we used the date of baseline participation in Gesundheit 65+ as the temporal reference and stratified our multivariable regression model by participation date in “before January 2022” vs. “after December 2021” (Supplementary table S2). To evaluate the robustness of our findings we used different definitions for the outcome variable (including the people that reported “yes, probably, not confirmed by a test” in the sample reporting a SARS-CoV-2 infection) (Supplementary table S4), and different definition for the immunization variable (receiving only one dose of the vaccine) (Supplementary table S3).

We calculated the variance inflation factor to assess for multicollinearity (Supplementary Table S1). Model specification was assessed using the link test. Design effect estimates (DEFF) for each model coefficient were obtained to assess variance inflation due to clustering. DEFF for model coefficients ranged from 1.08 to 2.47 (Supplementary table S5), indicating that the complex survey design, particularly clustering, increased variance and reduced precision for several estimates. The predictors with the highest DEFF included not having double vaccination (DEFF = 2.47), multimorbidity (DEFF = 2.40), and low education level (DEFF = 2.06), suggesting these variables were more affected by within-cluster similarity.

For our sensitivity analyses we ran additional multivariable logistic regressions with the different variable definitions (Supplementary table S3 and S4). To assess for potential reverse causality, we ran a multivariable logistic regression where the outcome was receiving any kind of support at home (no support/informal support or home care) and infection status was an exposure variable (Supplementary table S6).

## Supplementary Information

Below is the link to the electronic supplementary material.

Supplementary Material 1

## Data Availability

The authors state that there are some restrictions on access to the data on which the results are based. The dataset cannot be made publicly available because the informed consent of the study participants does not cover making the data publicly available. The dataset underlying the results is archived at the Research Data Centre of the Robert Koch Institute and can be accessed by researchers on reasonable request. The data can be accessed on site at the Secure Data Centre of the Research Data Centre of the Robert Koch Institute. Requests can be sent by e-mail to fdz@rki.de.
