# Supplementary material for: Nationwide cross-sectional study results on long-term care and SARS-CoV-2 infection among older adults in Germany during the COVID-19 pandemic
Source: Sci Rep. 2026 Feb 2;16:4334. doi: 10.1038/s41598-026-37108-7 (PMC12865030; doi:10.1038/s41598-026-37108-7)
Supplement: Supplementary file 1 — Supplementary Material 1 [file 41598_2026_37108_MOESM1_ESM.docx]

**Nationwide cross-sectional study results on long-term care and SARS-CoV-2 infection among older adults in Germany during the COVID-19 pandemic**

**Supplementary information**

Ana Magdalena Ordonez-Cruickshank^1,2^ (corresponding author), Hannelore Neuhauser^1^, Arina Zanuzdana^3^, Christina Poethko-Müller^1^, Beate Gaertner**^1^, Judith Fuchs**^1^ (additional corresponding author)

**shared last authorship

1 Department of Epidemiology and Health Monitoring, Robert Koch Institute, Berlin, Germany

2 Charité – Universitätsmedizin Berlin, corporate member of Freie Universität Berlin and Humboldt- Universität zu Berlin, Germany

3 Department of Infectious Disease Epidemiology, Robert Koch Institute, Berlin, Germany

Contact information:

E-mail: ana-magdalena.ordonez-cruickshank@charite.de

E-mail: fuchsj@rki.de

Address: Robert Koch Institute, Nordufer 20, 13353 Berlin, Germany

Contents

[Variance inflation factor 2](#_Toc219571709)

[Sensitivity analyses 3](#_Toc219571710)

[1. Questionnaire date 3](#_Toc219571711)

[2. Different immunization definition 4](#_Toc219571712)

[3. Different outcome definition 5](#_Toc219571713)

[Design effects 6](#_Toc219571714)

[Reverse causality analysis 7](#_Toc219571715)

# Variance inflation factor

Supplementary table S1. Variance inflation factor for variables included in the multivariable logistic regression analysis of risk factors for self-reported SARS-CoV-2 infections among people living in private households

| Variables | VIF |
| --- | --- |
| **Sociodemographic risk factors** |  |
| Age group in years (ref. 65-74) |  |
| 75-84 | 1.24 |
| 85+ | 1.41 |
| Sex (ref. male) |  |
| Female | 1.10 |
| Level of education (ref. high) |  |
| Low | 2.35 |
| Middle | 2.26 |
| Municipality size (ref. rural) |  |
| Small town | 2.06 |
| Medium town | 1.92 |
| City | 1.98 |
| **Health related risk factors** |  |
| Multimorbidity  (ref. no multimorbidity) | 1.07 |
| Smoking (ref. not smoking) | 1.06 |
| **Personal contact related risk factors** |  |
| Type of support (ref. no support) |  |
| Informal support | 1.22 |
| Home care | 1.23 |
| Not living alone (ref. living alone) | 1.12 |
| Participated in work/social activities (ref. no work/social activities) | 1.16 |
| Received in-person visits  (ref. no in-person visits) | 1.05 |
| **Immunization** |  |
| Not having double vaccination (ref. Double vaccination) | 1.03 |

Notes: VIF= Variance inflation factor; A link test indicated no model misspecification, as the squared predicted term was not significant (p=0.397), while the predicted term was statistically significant (p=0.011)

# Sensitivity analyses

## Questionnaire date

Supplementary table S2. Multivariable logistic regression analyses of factors associated with self-reported SARS-CoV-2 infections among people living in private households at the time of the baseline wave of the Gesundheit 65+ study stratified by date of study participation

|  | **June-December 2021 (n=1967)** | | **January-April 2022 (n=1094)** | |
| --- | --- | --- | --- | --- |
|  | OR (95% CI) | p-value* | OR (95% CI) | p-value* |
| **Sociodemographic risk factors** |  |  |  |  |
| Age group in years (ref. 65-74) |  |  |  |  |
| 75-84 | 2.08 (0.73-5.95) | 0.171 | 0.69 (0.34-1.39) | 0.297 |
| 85+ | 3.00 (1.01-8.92) | 0.047 | 0.36 (0.09-1.46) | 0.150 |
| Sex (ref. male) |  |  |  |  |
| Female | 1.09 (0.46-2.56) | 0.843 | 0.82 (0.45-1.49) | 0.514 |
| Level of education (ref. high) |  |  |  |  |
| Low | 0.58 (0.19-1.79) | 0.337 | 1.01 (0.40-2.59) | 0.978 |
| Middle | 0.37 (0.13-1.08) | 0.069 | 1.67 (0.66-4.24) | 0.281 |
| Municipality size (ref. rural) |  |  |  |  |
| Small town | 2.16 (0.67-6.99) | 0.197 | 1.08 (0.28-4.16) | 0.913 |
| Medium town | 1.18 (0.41-3.38) | 0.757 | 0.99 (0.17-5.67) | 0.991 |
| City | 2.50 (0.72-8.69) | 0.147 | 1.13 (0.29-4.32) | 0.860 |
| **Health related risk factors** |  |  |  |  |
| Multimorbidity  (ref. no multimorbidity) | 2.02 (0.84-4.87) | 0.116 | 1.27 (0.46-3.53) | 0.644 |
| Smoking (ref. not smoking) | 0.09 (0.02-0.51) | 0.007 | 0.52 (0.13-2.07) | 0.348 |
| **Personal contact related risk factors** | |  |  |  |
| Type of support (ref. no support) | |  |  |  |
| Informal support | 0.60 (0.25-1.45) | 0.256 | 2.07 (0.74-5.79) | 0.162 |
| Home care | 1.32 (0.40-4.38) | 0.644 | 0.49 (0.12-2.10) | 0.336 |
| Not living alone (ref. living alone) | 2.10 (0.89-4.94) | 0.091 | 1.74 (0.71-4.25) | 0.221 |
| Participated in work/social activities (ref. no work/social activities) | 0.76 (0.35 - 1.67) | 0.491 | 1.14 (0.57-2.28) | 0.716 |
| Received in-person visits  (ref. no in-person visits) | 5.91 (1.44 - 24.20) | 0.014 | 1.13 (0.45-2.84) | 0.794 |
| **Immunization** | |  |  |  |
| Not having double vaccination (ref. Double vaccination) | 23.22 (9.82-54.90) | <0.001 | 5.63 (1.25-25.43) | 0.025 |

Notes: *Wald test; OR = odds ratio; 95% CI = 95% confidence interval; ref. = reference group

## Different immunization definition

Supplementary table S3. Logistic regression analyses of factors associated with self-reported SARS-CoV-2 infections at the time of the baseline wave of the Gesundheit 65+ study among participants living in private households (With the variable immunization meaning being vaccinated at least once)

|  | **Bivariate analysis** |  | **Multivariable analysis** (n=3061) | |
| --- | --- | --- | --- | --- |
|  | OR (95% CI) | p-value* | OR (95% CI) | p-value* |
| **Sociodemographic risk factors** |  |  |  |  |
| Age group in years (ref. 65-74) |  |  |  |  |
| 75-84 | 0.89 (0.51-1.55) | 0.686 | 1.03 (0.56-1.88) | 0.932 |
| 85+ | 1.27 (0.60-2.68) | 0.537 | 1.05 (0.50-2.21) | 0.904 |
| Sex (ref. male) |  |  |  |  |
| Female | 1.06 (0.66-1.71) | 0.799 | 1.07 (0.62-1.83) | 0.812 |
| Level of education (ref. high) |  |  |  |  |
| Low | 0.80 (0.38-1.69) | 0.563 | 0.83 (0.37-1.85) | 0.638 |
| Middle | 0.87 (0.40-1.88) | 0.716 | 0.93 (0.41-2.13) | 0.870 |
| Municipality size (ref. rural) |  |  |  |  |
| Small town | 1.65 (0.74-3.67) | 0.216 | 1.87 (0.81-4.32) | 0.143 |
| Medium town | 1.14 (0.44-2.96) | 0.784 | 1.19 (0.47-2.97) | 0.714 |
| City | 1.73 (0.74-4.01) | 0.202 | 2.07 (0.87-4.91) | 0.099 |
| **Health related risk factors** |  |  |  |  |
| Multimorbidity  (ref. no multimorbidity) | 1.50 (0.75-3.02) | 0.253 | 1.42 (0.67-2.99) | 0.354 |
| Smoking (ref. not smoking) | 0.34 (0.11-1.12) | 0.076 | 0.36 (0.11-1.23) | 0.103 |
| **Personal contact related risk factors** | |  |  |  |
| Type of support (ref. no support) | | |  |  |
| Informal support | 1.18 (0.63-2.20) | 0.603 | 1.01 (0.50-2.04) | 0.980 |
| Home care | 1.72 (0.55-5.35) | 0.347 | 1.45 (0.56-3.75) | 0.436 |
| Not living alone (ref. living alone) | 1.53 (0.87-2.68) | 0.139 | 1.82 (0.96-3.47) | 0.067 |
| Participated in work/social activities (ref. no work/social activities) | 0.85 (0.51-1.40) | 0.509 | 0.87 (0.52-1.45) | 0.580 |
| Received in-person visits  (ref. no in-person visits) | 1.62 (0.70-3.78) | 0.261 | 2.53 (1.00-6.39) | 0.050 |
| **Immunization** | |  |  |  |
| Not having at least 1 vaccine dose (ref. having at least 1 vaccine dose) | 5.76 (2.44-13.59) | <0.001 | 8.29 (3.46-19.84) | <0.001 |

Notes: *Wald test; OR = odds ratio; 95% CI = 95% confidence interval; ref. = reference group

## Different outcome definition

Supplementary table S4. Logistic regression analyses of factors associated with self-reported SARS-CoV-2 infections at the time of the baseline wave of the Gesundheit 65+ study among participants living in private households. (“yes, confirmed by a test” or “yes, probably, not confirmed by a test” were considered as report of infection)

|  | **Bivariate analysis** |  | **Multivariable analysis** (n=3094) | |
| --- | --- | --- | --- | --- |
|  | OR (95% CI) | p-value* | OR (95% CI) | p-value* |
| **Sociodemographic risk factors** |  |  |  |  |
| Age group in years (ref. 65-74) |  |  |  |  |
| 75-84 | 0.79 (0.49-1.28) | 0.339 | 0.84 (0.49-1.44) | 0.530 |
| 85+ | 1.05 (0.55-1.97) | 0.891 | 0.80 (0.42-1.53) | 0.504 |
| Sex (ref. male) |  |  |  |  |
| Female | 0.94 (0.62-1.43) | 0.781 | 0.90 (0.55-1.46) | 0.669 |
| Level of education (ref. high) |  |  |  |  |
| Low | 0.82 (0.43-1.57) | 0.554 | 0.81 (0.40-1.65) | 0.565 |
| Middle | 1.12 (0.60-2.09) | 0.708 | 1.18 (0.60-2.31) | 0.637 |
| Municipality size (ref. rural) |  |  |  |  |
| Small town | 1.09 (0.56-2.12) | 0.790 | 1.30 (0.60-2.83) | 0.498 |
| Medium town | 1.19 (0.59-2.41) | 0.621 | 1.33 (0.60-2.93) | 0.475 |
| City | 1.07 (0.53-2.15) | 0.847 | 1.27 (0.55-2.91) | 0.572 |
| **Health related risk factors** |  |  |  |  |
| Multimorbidity  (ref. no multimorbidity) | 1.57 (0.87-2.83) | 0.133 | 1.59 (0.86-2.94) | 0.137 |
| Smoking (ref. not smoking) | 2.50 (1.02-6.09) | 0.044 | 0.35 (0.13-0.93) | 0.036 |
| **Personal contact related risk factors** | |  |  |  |
| Type of support (ref. no support) | | |  |  |
| Informal support | 1.24 (0.73-2.10) | 0.415 | 1.32 (0.72-2.41) | 0.367 |
| Home care | 1.46 (0.53-3.99) | 0.463 | 1.40 (0.53-3.71) | 0.493 |
| Not living alone (ref. living alone) | 1.15 (0.72-1.83) | 0.566 | 1.28 (0.70-2.33) | 0.421 |
| Participated in work/social activities (ref. no work/social activities) | 0.91 (0.59-1.42) | 0.685 | 0.88 (0.56-1.40) | 0.593 |
| Received in-person visits  (ref. no in-person visits) | 1.62 (0.79-3.32) | 0.185 | 2.69 (1.20-6.07) | 0.017 |
| **Immunization** | |  |  |  |
| Not having double vaccination (ref. Double vaccination) | 5.25 (2.82-9.74) | <0.001 | 6.93 (3.60-13.35) | <0.001 |

Notes: *Wald test; OR = odds ratio; 95% CI = 95% confidence interval; ref. = reference group

# Design effects

Supplementary table S5. Design effects for variables included in the multivariable logistic regression analysis of factors associated with self-reported SARS-CoV-2 infections among people living in private households

| Variables | Design effects |
| --- | --- |
| **Sociodemographic risk factors** | |
| Age group in years (ref. 65-74) |  |
| 75-84 | 1.73 |
| 85+ | 1.22 |
| Sex (ref. male) | 1.60 |
| Female | 1.60 |
| Level of education (ref. high) |  |
| Low | 2.06 |
| Middle | 1.95 |
| Municipality size (ref. rural) |  |
| Small town | 1.80 |
| Medium town | 1.89 |
| City | 1.83 |
| **Health related risk factors** | |
| Multimorbidity (ref. no multimorbidity) | 2.40 |
| Smoking (ref. not smoking) | 1.57 |
| **Personal contact related risk factors** | |
| Type of support (ref. no support) |  |
| Informal support | 1.99 |
| Home care | 1.54 |
| Not living alone (ref. living alone) | 1.58 |
| Participated in work/social activities (ref. no work/social activities) | 1.66 |
| Received in-person visits (ref. no in-person visits) | 1.08 |
| **Immunization** | |
| Not having double vaccination (ref. Double vaccination) | 2.47 |

# Reverse causality analysis

Supplementary table S6. Multivariable logistic regression analysis of factors associated with receiving any type of support at the time of the baseline wave of the Gesundheit 65+ study among participants living in private households (n= 3,061)

|  | **OR (95% CI)** | **p-value*** |
| --- | --- | --- |
| **Sociodemographic risk factors** |  |  |
| Age group in years (ref. 65-74) |  |  |
| 75-84 | 2.11 (1.62-2.77) | <0.001 |
| 85+ | 8.48 (6.37-11.30) | <0.001 |
| Sex (ref. male) |  |  |
| Female | 1.56 (1.20-2.03) | 0.001 |
| Level of education (ref. high) |  |  |
| Low | 2.10 (1.54-2.86) | <0.001 |
| Middle | 1.70 (1.20-2.40) | 0.003 |
| Municipality size (ref. rural) |  |  |
| Small town | 0.73 (0.51-1.06) | 0.098 |
| Medium town | 0.68 (0.47-0.99) | 0.043 |
| City | 0.81 (0.54-1.20) | 0.289 |
| **Health related risk factors** |  |  |
| Multimorbidity (ref. no multimorbidity) | 3.45 (2.48-4.79) | <0.001 |
| Smoking (ref. not smoking) | 0.82 (0.52-1.30) | 0.401 |
| **Personal contact related risk factors** | |  |
| Not living alone (ref. living alone) | 0.94 (0.72-1.24) | 0.668 |
| Participated in work/social activities (ref. no work/social activities) | 0.45 (0.34-0.58) | <0.001 |
| Received in-person visits (ref. no in-person visits) | 1.07 (0.74-1.56) | 0.261 |
| **COVID-19 related variables** | |  |
| Not having double vaccination (ref. Double vaccination) | 0.94 (0.57-1.54) | 0.799 |
| Self-reported positive SARS-CoV-2 test (ref. self-report of not having a previous SARS-CoV-2 infection) | 1.11 (0.58-2.11) | 0.746 |

Notes: *Wald test; OR = odds ratio; 95% CI = 95% confidence interval; ref. = reference group
